# Subclinical patterns of cardiac involvement by transthoracic echocardiography in individuals with mild initial COVID-19

**DOI:** 10.1038/s41598-025-85221-w

**Published:** 2025-01-30

**Authors:** Anastasia Shchendrygina, Mame Madjiguène Ka, Carlos Rodriguez, Safaa Alsoufi, Jedrzej Hoffmann, Parveen Kumar, Maria Ludovica Carerj, Byambasuren Vanchin, Niels Holm, Argyro Karyou, Mijidsuren Ganbat, Eike Nagel, Valentina O. Puntmann

**Affiliations:** Institute for Experimental and Translational Cardiovascular Imaging, DZHK Centre for Cardiovascular Imaging, Goethe University Frankfurt, Frankfurt am Main, Germany

**Keywords:** Transthoracic echocardiography, PASC, Subclinical, Diastolic function, Global longitudinal strain, Cardiovascular diseases, Cardiology, Medical research, Preclinical research

## Abstract

The aim of this study was to evaluate the subclinical patterns and evolution of cardiac abnormalities via transthoracic echocardiography (TTE) in patients with mild initial COVID-19 illness. A total of 343 infected individuals (163 males; age 44 (interquartile range, IQR 35–52) years) years) underwent serial TTE assessments at a median of 109 (interquartile range (IQR), 77–177) and 327 (276–379) days after infection. Compared with those of non-COVID-19-infected controls (*n* = 94, male *n* = 49), baseline systolic (LVEF, TAPSE) and diastolic function (eʹ, aʹ, E/eʹ) were significantly different in infected participants (*p* < 0.05 for all). Compared with baseline assessments, there was a reduction in global longitudinal strain (GLS) and an increase in the E wave, E/A ratio and E/eʹ at follow-up. At baseline, symptomatic participants had a lower LVEF and TAPSE and increased IVRT, eʹ and E/eʹ. At follow-up, symptomatic patients had a lower LV end-diastolic diameter (LVEDd). Symptoms were independently associated with E/eʹ at baseline (OR (95% CI) 1.45 (1.12–1.87), *p* = 0.005). Symptoms at follow-up were associated with LVEDd, measured either at baseline (OR: 0.91 (0.86, 0.96), *p* < 0.001) or follow-up (OR (95% CI) 0.91 (0.86–0.96), *p* < 0.001). There were significant associations for GLS and troponin and E/eʹ with CRP and NTproBNP at baseline. In the present cohort of COVID-19-infected individuals with mild initial illness, echocardiographic measurements revealed significant yet subclinical differences in systolic and diastolic function compared with controls, as well as between individuals with cardiac symptoms and those without. All the measured differences were small in magnitude and thus unlikely to be detectable clinically at the individual level.

## Introduction

Cardiovascular inflammatory involvement is an increasingly recognized sequela of COVID-19 disease, both acutely and after resolution of the viral illness^1^. The observational studies detail a high prevalence of symptoms, including shortness of breath, chest pain, excessive tachycardia, progressive effort intolerance and increased blood pressure (reviewed in^2^). Women have a greater predisposition for persistent symptoms, including the development of chronic fatigue syndrome^3–5^. The outcomes of cardiac imaging studies include signs of deterioration of both right (RV) and left ventricular (LV) function in a substantial proportion of patients, as well as an overall higher rate of cardiac complications and poor prognosis^6–10^. Studies using cardiovascular magnetic resonance revealed ongoing subclinical inflammatory involvement, which, at least in part, is related to persistent symptoms^5^. Echocardiographic changes related to cardiac involvement in previously healthy subjects with mild acute illness, including the evolution of abnormalities over time, remain less well explored. We report the outcomes of serial assessments by transthoracic echocardiography (TTE) in individuals with initially mild COVID-19.

## Methods

### Study population

This was a prospective single-centre observational cohort multimodality imaging study of individuals at risk of possible subclinical cardiac involvement, but there was no formal clinical indication for CMR imaging (Impression Study, NCT04444128). The study design and partial results have been reported previously^5^. In brief, the primary objective of impression study, initiated in 2017, was to investigate the longitudinal changes of subclinical development of cardiovascular involvement prior to development of overt structural heart disease in conditions that may potentially predispose to development of heart disease in due course. The research objective of the Impression study was to draw on the deeper pathophysiological signatures in health and early disease by means of comprehensive cardiac imaging and tissue characterisation. An additional objective was to define ‘health’ with a strong emphasis on comparisons of various MRI sequences including normal values and reproducibility in subjects with no known cardiac disease or comorbidities, who were also included and serve as controls. The COVID substudy started in March 2020 to allow investigations of the context of a new disease. A total of 343 subjects who recovered from COVID-19 infection at home were included in the present analysis. The participants underwent baseline assessments after a minimum of 4 weeks from the initial diagnosis of COVID-19 between April 2020 and October 2021 and a follow-up examination after a minimum of 4 months from baseline. The inclusion and exclusion criteria were previously reported in detail^5^. In brief, the subjects were subjected to laboratory-confirmed COVID-19 infection via reverse-transcriptase polymerase chain reaction (RT‒PCR) assays performed on nasopharyngeal swabs. The exclusion criteria were severe acute COVID-19 illness requiring hospitalization; previously established cardiovascular disease (prior diagnosis of hemodynamically significant coronary artery disease; history of revascularization or cardiovascular device implantation; structural heart disease; heart failure; cardiomyopathy; significant valvular disease (≥ grade III); known liver or kidney disease; uncontrolled diabetes; other significant endocrine, rheumatological or oncological conditions; receiving any treatment; and not willing to provide informed consent or to participate in long-term follow-up. Clinically and laboratory euthyroid patients receiving thyroxine supplements were not excluded. Control group consisted of subjects with identical inclusion and exclusion criteria, recruited prior to 2020 with similar distributions for age, sex and cardiovascular risk factors.

All participants underwent standardized clinical assessments, including questionnaires, cardiac imaging, and blood sampling, prior to imaging assessments on the same day. The presence of symptoms was defined as cardiac symptoms (present or absent), including chest pain, dyspnoea, palpitations and syncope, which were recorded and graded using standardized questionnaires, reported previously^5^. Chest discomfort was graded using a modified chest discomfort scale based on the Canadian Chest Pain Scale, not limited to the typical anginal type of chest pain, but also included, deep, dull, pulling, burning or sharp chest discomfort or tightness, radiating into the neck, back, shoulders or arms. To differentiate this from precordial catch symptoms and abdominal stiches, the criterion for chest pain had to last more than 10 min to qualify. Dyspnoea was graded using modified Medical Research Council Dyspnoea Severity Score. Palpitations and syncope were classified as present or absent.

Blood samples were processed via standardized commercially available test kits for the analysis of high-sensitivity troponin T (hsTnT) and N-terminal pro–B-type natriuretic peptide (NTproBNP; Elecsys 2010; Roche). Demographics, assessments of symptoms, clinical biomarkers and results of cardiac MRI were reported previously^5^. The study was approved by the institutional ethics committee (Ethics Committee of University Hospital Frankfurt; Germany). All procedures were performed in accordance with the Declaration of Helsinki and the International Conference on Harmonization of Good Clinical Practice. All patients provided written informed consent.

### Transthoracic echocardiography

**Fig. 1:**
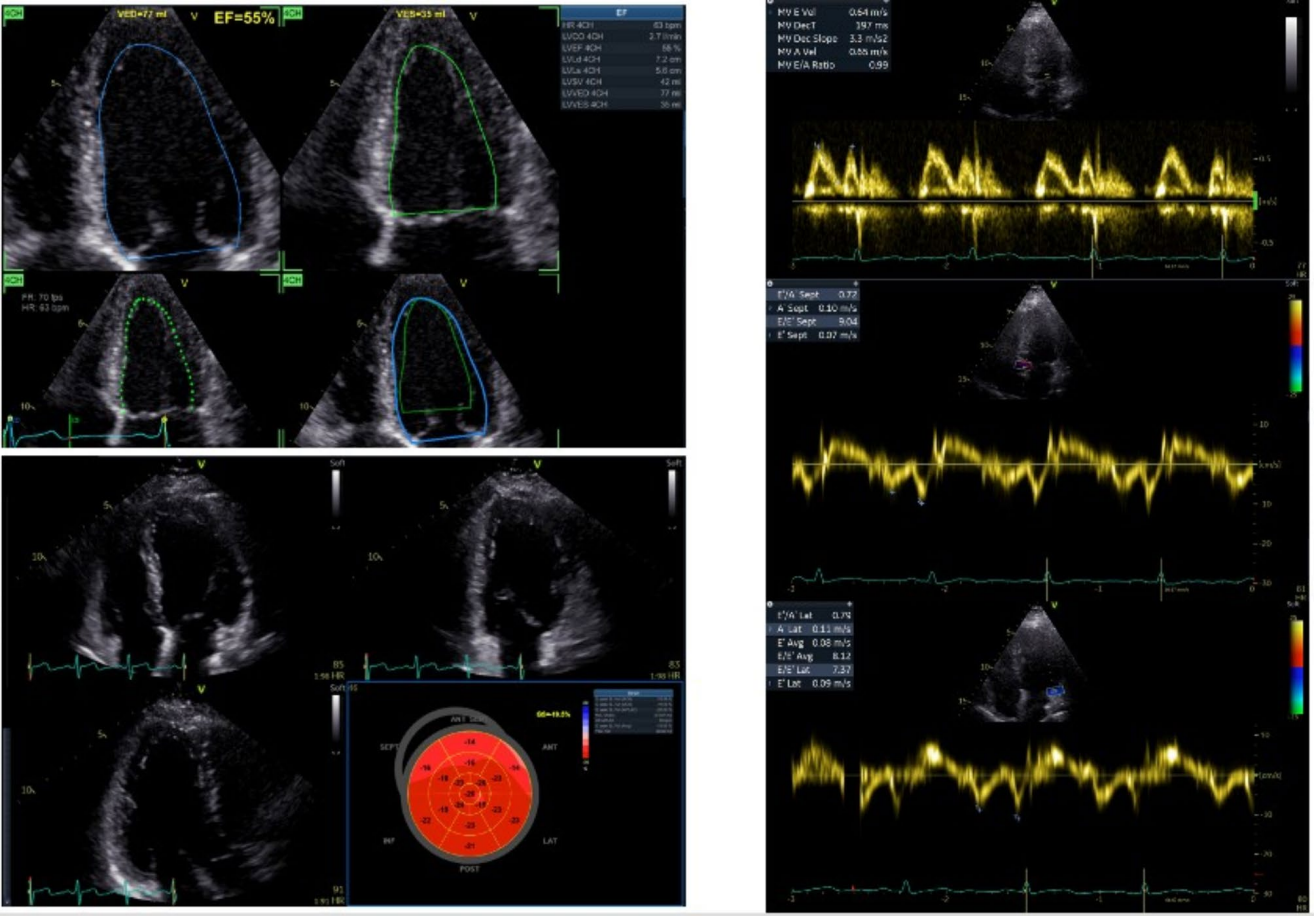
Representative images of echocardiographic acquisition.

TTE was performed via a commercially available system (General Electrics, GE Vivid E95), and all measurements were performed in line with the recommendations of the World Alliance Societies of Echocardiography^11–13^. All the images were analysed offline with EchoPAC version 203 (GE, Vingmed Ultrasound AS) by trained cardiologists who were blinded to the clinical information. Echocardiographic parameters included assessments of cardiac structure, systolic and diastolic function, and cardiac flow. LV ejection fraction (LVEF) was measured via Simpson’s biplane method. Pulsed-wave tissue Doppler was used for assessing peak transmitral flow velocities (E-wave, A-wave) and their respective ratios (E/A). Tissue Doppler imaging was used for assessment of peak early diastolic lateral and septal e′-velocity. The average E/e′ was calculated as an average of the septal and lateral measurements. RV function was measured via the tricuspid annular plane systolic excursion (TAPSE) in M-mode. Valvular hemodynamic parameters were also assessed. Myocardial speckle strain analysis was performed offline via 2D speckle tracking strain analysis in standard apical views (two-, three-, and four-chamber views) with automatic contour tracking of the LV endocardium and manual correction whenever necessary. Global longitudinal strain (GLS) was calculated by averaging the mean peak GLS values of 16 LV segments from all three apical views^11,14^; GLS is presented in percentages as absolute (positive) values. Representative images are included in Fig. 1.

### Statistical analysis

The normality of distributions was tested with the Kolmogorov‒Smirnov statistic. Baseline categorical variables are shown as numbers and percentages; the differences between the groups were computed with Fischer’s exact test for proportions. Continuous variables are presented as medians and interquartile ranges (IQRs), and differences between groups were compared via nonparametric tests. Relationships were analysed via regression analyses. Associations with symptoms were determined via univariate and multivariate binary logistic regression analyses (forward), adjusting for multiple testing to reduce the risk of type I error using false discovery rate. For all analyses, p values < 0.05 were considered significant. Reliability was assessed via the Bland‒Altman method for interobserver and intraobserver differences in 33 randomly selected cases. Interobserver and intraobserver variability were assessed via intraclass correlation coefficients (ICCs) via a two-way mixed model. Statistical analysis was performed via JASP 0.18.3 and r-studio Version 2023.12.0.

## Results

A total of 343 infected individuals (163 males, (48%); age 44 (interquartile range, IQR 35–52) years) years) underwent serial TTE assessments at a median of 109 (interquartile range (IQR), 77–177) days after infection. Follow-up echocardiograms were available for 304 participants, and 327 (276–379); 39 subjects either refused a repeat echo (*n* = 19), or images were of insufficient quality for LVEF postprocessing (*n* = 20). The detailed participant characteristics and symptom scores were reported previously^5^. Compared to non-COVID controls, infected individuals had higher resting heart rate, systolic and diastolic blood pressure at baseline, as reported previously^5^. None of the participants had increased LV wall thickness, LVEF < 45% or overt structural heart disease. No cases of moderate or severe valvular pathology were identified.

### Echocardiographic findings at baseline compared with those of controls

Compared with non-COVID-19-infected controls (*n* = 94, 49 males, 52%), infected individuals had higher resting heart rates and systolic and diastolic blood pressures, as reported previously^5^, as well as mildly higher hsTNT. Baseline systolic (LVEF, TAPSE) and diastolic function (eʹ, aʹ, E/eʹ) were significantly different among the infected participants (*p* < 0.05 for all, Table 1).

**Table 1:** Overview of participants’ characteristics at baseline and follow-up.

Characteristic	Controls, *N* = 94	Cases baseline, *N* = 343	Sig. (*p* value)	Cases follow-up, *N* = 304	Sig. (*p* value)
Age (years)	41.50 [28.25, 49.00]	44.00 [34.50, 52.00]	0.051	44.50 [35.00, 53.00]	0.54
Gender (male)	49 (52%)	163 (48%)	0.43	143 (47%)	0.80
BMI (kg/m^2^)	24.12 [22.61, 26.28]	24.42 [22.24, 27.42]	0.70	24.6 [22.5, 27.5]	0.60
Heart rate (bpm)	68.00 [60.25, 73.00]	68.00 [62.00, 76.00]	0.04	67.00 [60.00, 74.00]	0.01
Systolic BP (mmHg)	128.00 [115.00, 138.00]	130.00 [120.00, 142.00]	0.01	126.0 [117.0, 138.0]	0.008
Diastolic BP (mmHg)	79.00 [72.00, 87.00]	82.00 [77.00, 90.00]	0.002	82.0 [74.0, 89.5]	0.15
CRP (mg/dl)	0.08 [0.04, 0.19]	0.08 [0.04, 0.18]	0.72	0.1 [0.1, 0.2]	0.52
HsTnT (pg/ml)	4.00 [3.00, 5.00]	4.07 [3.00, 5.77]	0.048	3.7 [3.0, 5.7]	0.06
NTproBNP(pg/ml)	36.90 [20.50, 65.70]	44.20 [23.55, 79.05]	0.17	41.0 [21.6, 75.0]	0.47
Echo parameters					
LVEF (%)	59.50 [56.00, 64.00]	56.00 [55.00, 61.00]	< 0.001	57.00 [55.00, 62.00]	0.15
LVEDd (mm)	48.00 [44.00, 52.00]	48.00 [44.00, 51.00]	0.58	47.00 [44.00, 51.00]	0.91
LVEDs (mm)	33.00 [30.00, 36.00]	32.00 [28.00, 36.00]	0.19	32.00 [29.00, 36.00]	0.41
RWT (mm)	0.34 [0.30, 0.42]	0.36 [0.31, 0.42]	0.92	0.37 [0.32, 0.42]	0.13
MAPSE (mm)	17.00 [16.00, 20.00]	17.00 [15.00, 19.00]	0.25	17.00 [14.00, 19.00]	0.24
GLS (%)	19.40 [18.12, 21.67]	19.50 [18.20, 21.31]	0.60	18.92 [17.46, 20.40]	0.002
E wave (m/s)	0.76 [0.66, 0.84]	0.74 [64, 87]	0.50	0.82 [0.69, 0.92]	< 0.001
A wave (m/s)	0.62 [0.51, 0.74]	0.61 [0.51, 0.71]	0.28	0.60 [0.48, 0.73]	0.76
E/A ratio	1.15 [0.90, 1.48]	1.23 [0.98, 1.49]	0.22	1.28 [1.0-1.68]	0.02
DecT (ms)	214 [183, 246]	199 [172, 231]	0.01	198.00 [163.00, 226.80]	0.49
IVRT (ms)	97.00 [76.00, 109.50]	90.00 [76.00, 104.00]	0.97	93.0 [80.0, 118.0]	0.02
Eʹsp (cm/s)	9.50 [8.00, 12.00]	9.00 [8.00, 11.00]	0.27	9.0 [8.0, 11.0]	0.16
Eʹlat (cm/s)	13.00 [11.00, 15.75]	11.00 [9.00, 14.00]	0.004	11.0 [9.0, 13.0]	0.13
Aʹsp (cm/s)	9.00 [7.50, 11.00]	8.00 [7.00, 10.00]	0.03	9.00 [7.00, 10.00]	0.04
Aʹlat (cm/s)	10.00 [7.00, 11.00]	8.00 [7.00, 10.00]	0.02	8.00 [7.00; 10.00]	0.83
E/eʹ sp	7.61 [6.53, 8.54]	7.74 [6.43, 9.17]	0.08	8.24 [7.09, 10.18]	< 0.001
E/eʹlat	5.62 [4.79, 6.91]	6.53 [5.38, 7.88]	< 0.001	7.23 [5.90, 8.89]	< 0.001
E/eʹ (median)	6.53 [5.60, 7.59]	7.00 [6.00, 8.45]	< 0.001	7.78 [6.52, 9.31]	< 0.001
LA_PLAX (mm)	33.00 [29.00, 37.00]	32.00 [29.00, 37.00]	0.66	34.00 [30.00, 38.00]	0.03
LAVI (cm^3^/m^2^)	20.00 [17.00, 25.00]	21.00 [18.00, 26.00]	0.43	22.00 [19.00, 25.50]	0.62
RVEDD (mm)	29.00 [26.00, 33.00]	30.00 [27.00, 33.00]	0.38	30.00 [27.00, 34.00]	0.62
TAPSE (mm)	24.00 [22.00, 27.00]	23.00 [21.00, 26.00]	0.02	23.00 [21.00, 26.00]	0.91
RA_4CH (cm^2^)	16.50 [14.00, 20.00]	15.00 [13.00, 18.75]	0.21	15.00 [13.00, 19.00]	0.34
TR Vmax (m/sec)	2.17 [1.85, 2.30]	1.90 [1.57, 2.19]	0.13	1.90 [1.60, 2.17]	0.98

Median (IQR). Comparisons were made between controls and cases at the baseline, and cases between baseline vs. follow-ups. Group comparisons were examined using nonparametric tests’ p values < 0.05 were considered significant. GLS presented as in absolute (positive) numbers.

Significant values are given in bold.

### Echocardiographic findings on follow-up evaluation in comparison with baseline assessment

Compared with those at baseline (Table 1), heart rate and systolic blood pressure were lower, whereas diastolic blood pressure remained similar to that at baseline. There was a reduction in GLS (*p* = 0.002) and an increase in the E wave, E/A ratio, aʹ septal, E/eʹ and IVRT, as well as the LA diameter at follow-up (*p* < 0.05 for all).

### Comparison of symptomatic vs. asymptomatic patients at baseline and follow-up

There were significant differences between symptomatic participants (*n* = 249) and those without symptoms (*n* = 94) at baseline (Table 2). Symptomatic participants had a significantly greater heart rate (bpm 65 [IQR: 59, 75] vs. 68 [60, 76], *p* = 0.03), lower LVEF and TAPSE, and higher IVRT, eʹ septal and lateral, E/eʹ, and TR Vmax. At follow-up (Table 3), heart rate (bpm, 65 [60, 72] vs. 68 [60, 76], *p* = 0.02), NTproBNP (pg/ml, 36 [17–69], vs. 48 [25–82], *p* = 0.02), and LVEDd and LVEDs were significantly different between symptomatic participants (*n* = 168) and those without symptoms (*n* = 134).

**Table 2:** Echo parameters of patients with and without cardiovascular symptoms at baseline.

Characteristic	Sy−, *N* = 94	Sy+, *N* = 249	*p* value
LVEF (%)	57.5 [55.0, 62.0]	56.0 [55.0, 60.0]	0.05
LVEDd (mm)	48.5 [44.0, 52.8]	48.0 [44.0, 50.0]	0.232
LVEDs (mm)	32.0 [28.0, 36.8]	32.0 [28.0, 36.0]	0.482
RWT (mm)	0.36 [0.31, 0.42]	0.35 [0.31, 0.42]	0.59
MAPSE (mm)	18.00 [16.00, 20.00]	17.00 [15.00, 19.00]	0.06
GLS (%)	19.98 [18.33, 21.53]	19.45 [18.15, 21.26]	0.32
E wave (m/s)	0.71 [0.63, 0.85]	0.75 [0.65, 0.87]	0.18
A wave (m/s)	0.60 [0.51, 0.84]	0.61 [0.51, 0.87]	0.66
E/A ratio	1.25 [0.96, 1.44]	1.23 [0.99, 1.50]	0.75
DecT (ms)	202 [174, 233]	195 [172, 227]	0.43
IVRT (ms)	86 [72, 100]	90 [79, 106]	0.04
Eʹsp (cm/s)	10.00 [9.00, 12.00]	9.00 [8.00, 11.00]	< 0.001
Eʹlat (cm/s)	12.00 [10.00, 15.00]	11.00 [9.00, 14.00]	0.01
Aʹsp (cm/s)	8.00 [7.00, 10.00]	8.00 [7.00, 10.00]	0.94
Aʹlat (cm/s)	8.00 [7.00, 11.00]	8.00 [6.00, 10.00]	0.63
E/eʹsp	7.10 [6.09, 8.17]	8.00 [6.85, 9.44]	< 0.001
E/eʹlat	6.04 [4.85, 7.27]	6.83 [5.43, 8.21]	< 0.001
E/eʹ (median)	6.68 [5.34, 7.59]	7.27 [6.16, 8.71]	< 0.001
LA_PLAX (mm)	32.50 [30.00, 37.00]	32.00 [29.00, 37.50]	0.70
LAVI (cm^3^/m^2^)	21.63 [17.96, 26.51]	21.0 [17.96; 26.00]	0.77
RVEDD (mm)	30.50 [27.75, 34.0.]	29.00 [27.00, 33.00]	0.18
TAPSE (mm)	24.50 [21.00, 28.00]	23.00 [20.00, 26.00]	0.02
RA_4CH (cm^2^)	15.50 [13.00, 18.25]	15.00 [13.00, 18.75]	0.62
TR Vmax (m/s)	1.78 [1.49; 1.90]	2.00 [1.70; 2.30)	0.01

Median (IQR). Comparisons were made between symptomatic and asymptomatic cases at the baseline, group comparisons were examined using nonparametric tests’ p values < 0.05 were considered significant. GLS presented as in absolute (positive) numbers.

Significant values are given in bold.

**Table 3:** Echocardiographic parameters of patients with and without cardiovascular symptoms at follow-up.

Characteristic	Sy−, *N* = 136	Sy+, *N* = 168	*p* value
LVEF (%)	57.0 [54.0, 62.0]	57.0 [55.0, 62.0]	0.58
LVEDd (mm)	48.0 [45.0, 54.0]	47.0 [44.0, 49.0]	0.006
LVEDs (mm)	33.0 [30.0, 37.3]	32.0 [28.0, 35.0]	0.07
RWT (mm)	0.36 [0.31; 0.41]	0.35 [0.31; 0.43]	0.42
MAPSE (mm)	17.00 [15.00, 20.00]	17.00 [15.00, 19.00]	0.95
GLS (%)	18.62 [17.39, 20.32]	19.20 [17.67, 20.62]	0.19
E wave (m/s)	0.81 [0.71; 0.92]	0.82 [0.69; 0.92]	0.87
A wave (m/s)	0.58 [0.48; 0.72]	0.61 [0.49; 0.73]	0.38
E/A ratio	1.27 [0.99; 1.51]	1.22 [0.97; 1.47]	0.26
DecT (ms)	202 [166; 228]	196 [161; 221]	0.33
IVRT (ms)	93 [83; 121]	91 [79; 115]	0.51
Eʹsp (cm/s)	9.0.0 [8.00, 11.00]	9.00 [8.00, 11.00]	0.54
Eʹlat (cm/s)	11.00 [9.00, 13.00]	11.00 [9.00, 13.00]	0.45
Aʹsp (cm/s)	8.00 [7.00; 10.00]	9.00 [7.00; 10.00]	0.70
Aʹlat (cm/s)	8.00 [6.00; 10.00]	8.00 [7.00; 10.00]	0.18
E/eʹsp	8.25 [7.09, 10.17]	8.45 [7.10, 10.05]	0.86
E/eʹlat	7.18 [6.00, 8.76]	7.20 [5.78, 9.00]	0.78
E/eʹ average	7.62 [6.58, 9.23]	7.83 [6.46, 9.40]	0.76
LA_PLAX (mm)	34.00 [30.00, 38.76]	34.00 [30.00, 38.00]	0.74
LAVI (cm^3^/m^2^)	21.00 [17.93; 25.75]	21.00 [18.00, 26.00]	0.62
RVEDD (mm)	30.50 [28.00, 34.00]	29.00 [26.00, 34.00]	0.27
TAPSE (mm)	23.00 [21.00, 26.00]	23.0 [20.75, 26.00]	0.83
RA_4CH (cm^2^)	16.00 [13.00, 19.50]	15.00 [13.00, 19.00]	0.40
TR Vmax (m/s)	1.90 [1.40; 2.05]	2.00 [1.61; 2.190]	0.09

Comparisons were made between symptomatic and asymptomatic cases at the follow-up, group comparisons were examined using nonparametric tests’ p values < 0.05 were considered significant. GLS presented as in absolute (positive) numbers.

Significant values are given in bold.

### Analysis of relationships

The results of binary logistic regressions for predictive associations of variables with the presence of symptoms at the timepoint are presented in Table 4. At baseline, the symptoms were univariately associated with heart rate, LVEF, TAPSE, E/eʹ and TR Vmax. At follow-up, female sex, heart rate, NTproBNP, LVEDd, and LVEDs, which are continuous variables, were univariably associated with the presence of symptoms. Accounting for the multicollinearity of multiple variables in binary logistic regression (forward), the presence of symptoms at baseline was independently associated with E/eʹ at baseline (OR (95% CI) 1.45 (1.12, 1.87), *p* = 0.005). At follow-up, the symptoms were associated with LVEDd (OR (95% CI) 0.91 (0.86–0.96), *p* < 0.001) (Chi^2^ 14.78; *p* < 0.001). A prediction model for follow-up status using baseline measurements reiterated the strong role of LVEDd (OR 0.91 (0.86, 0.96), *p* < 0.001).

**Fig. 2:**
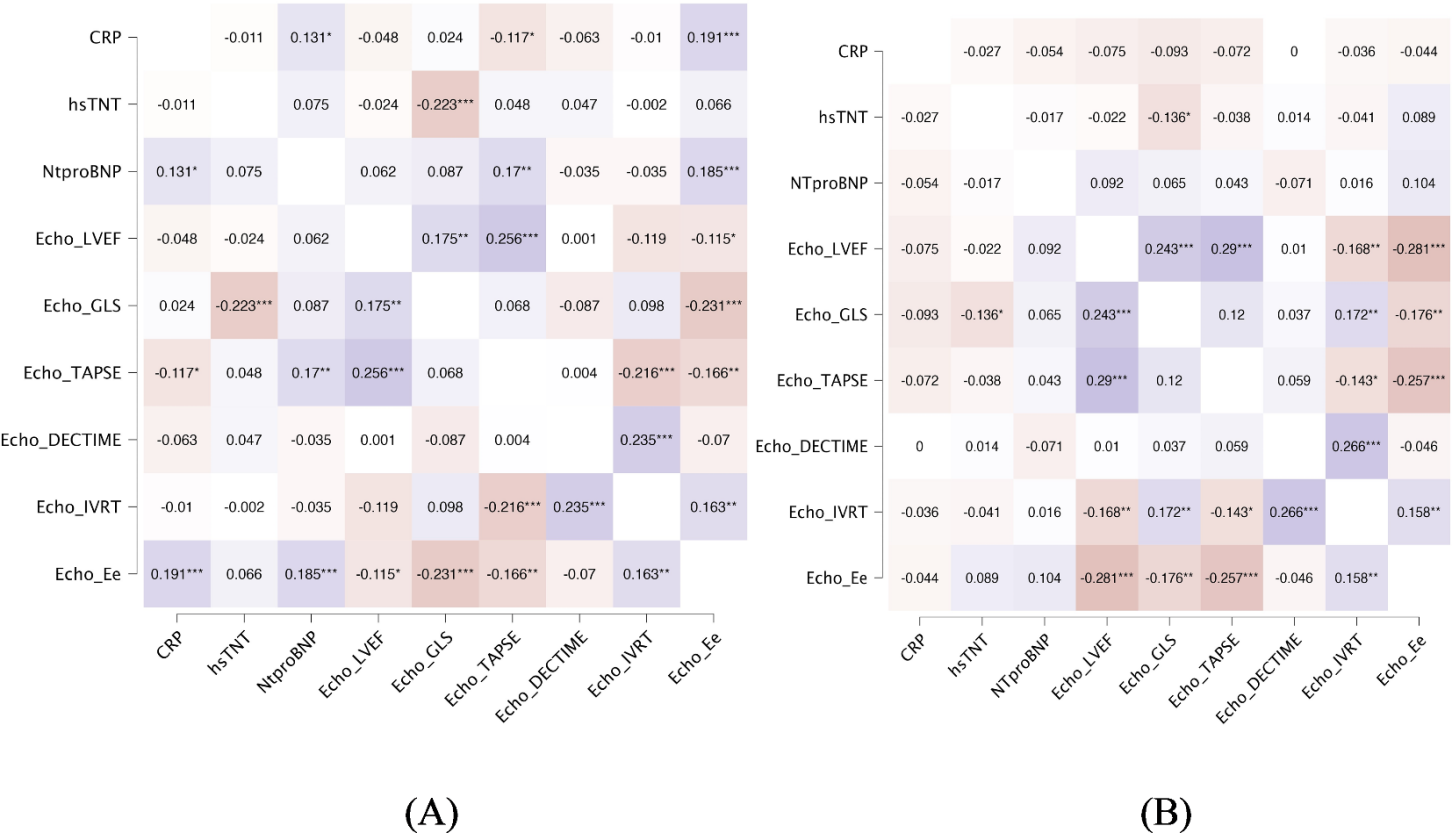
Heatmap of the associations between echocardiography variables and cardiac biomarkers (**A**) baseline, (**B**) follow-up and Spearman correlations (rho, p value).

The results of correlation between echo parameters with blood biomarkers at baseline and follow-up are shown in Fig. 2. There were significant associations for GLS and troponin and E/e’ with CRP and NTproBNP at baseline. There were no significant associations with biomarkers at follow-up. The intraobserver variability in 33 randomly selected cases for LVEF was − 0.54 (95% CL −1,6–0,57) (ICC) = 0.76), whereas the interobserver variability was 0,15± (95% CL −1,19–1,49) (ICC = 0.70). The intraobserver variability for GLS was − 0,33 (95% CI −0,1 to 0,77) (ICC = 0.77), whereas the interobserver variability was − 0,39 (95% CI −0,88 to 0,09) (ICC = 0.73).

**Table 4:** Results of uni- and multivariable binary logistic regression analyses for the prediction of cardiovascular symptoms at the timepoint, baseline (**A**) and follow-up (**B**); prediction of symptoms at follow-up based on baseline values (**C**).

	A. Baseline		B. Follow up		C. Baseline variables Follow-up status	
Univariable	OR (95%CI)	Sig. (p value)	OR (95%CI)	Sig. (p value)	OR (95%CI)	Sig. (p value)
Age (years)	1.00 (0.98, 1.02)	0.87	1.02 (1.00, 10.4)	0.15	1.02 (1.00, 1.04)	0.04
Gender (female)	1.54 (0.96, 2.49)	0.25	2.20 (1.39, 3.50)	< 0.001	2.41(1.56, 3.75)	< 0.001
Heart rate (ms)	1.03 (1.00, 1.05)	0.02	1.03 (1.0, 1.05)	0.02	1.02 (1.00, 1.05)	0.02
BP systolic (mmHg)	0.99 (0.98, 1.01)	0.27	0.99 (0.98, 1.01)	0.25	1.00 (0.98, 1.01)	0.45
BP diastolic (mmHg)	1.01 (0.99, 1.04)	0.22	1.02 (1.00, 1.04)	0.054	1.01 (1.0, 1.04)	0.15
CRP (mg/dl)	0.93 (0.31, 3.15)	0.90	0.95 (0.88–1.03)	0.25	1.59 (0.55, 4.98)	0.40
hsTNT (pg/ml)	0.97 (0.88, 1.07)	0.51	0.95 (0.88–1.03)	0.23	0.96 (0.88, 1.05)	0.38
NTproBNP (pg/ml)	1.00 (0.99, 1.00)	0.76	1.01 (1.00, 1.01)	0.02	1.00 (1.00, 1.01)	0.26
LVEF (%)	0.94 (0.89, 0.99)	0.02	1.01 (0.97, 1.05)	0.68	0.98 (0.93, 1.02)	0.34
LVEDd (mm)	0.97 (0.93, 1.01)	0.15	0.93 (0.89, 0.97)	< 0.001	0.92 (0.89, 0.96)	< 0.001
LVEDs (mm)	0.98 (0.95, 1.02)	0.37	0.96 (0.93, 1.00)	0.041	0.94 (0.91, 0.98)	< 0.001
RWT (mm)	0.48 (0.04, 6.30)	0.57	1.31 (0.13, 13.8)	0.43	1.95 (0.18, 22.5)	0.59
MAPSE (mm)	1.00 (0.97, 1.05)	0.96	1.00 (0.94, 1.06)	0.97	0.97 (0.91, 1.02)	0.21
GLS (%)	0.94 (0.85, 1.05)	0.27	1.06 (0.97, 1.17)	0.34	1.07 (0.97, 1.18)	0.19
E wave (m/s)	1.01 (0.99, 1.03)	0.23	1.00 (0.98, 1.02)	0.73	1.00 (0.99, 1.02)	0.81
A wave (m/s)	1.00 (0.99, 1.02)	0.63	1.01 (0.99, 1.02)	0.38	1.02 (1.00, 1.03)	0.03
E/A ratio	1.96 (0.66, 2.16)	0.55	0.74 (0.48, 1.12)	0.15	0.68 (0.40, 1.15)	0.15
DecT (ms)	1.00 (0.99, 1.00)	0.43	1.00 (0.99, 1.00)	0.29	0.99 (0.98, 1.00)	< 0.001
IVRT (ms)	0.99 (0.99, 1.00)	0.54	1.00 (0.99, 1.00)	0.60	0.99 (0.99, 1.00)	0.07
Eʹsp (cm/s)	0.87 (0.75, 1.02)	0.08	0.93 (0.88, 1.08)	0.61	0.93 (0.85, 1.02)	0.14
Eʹlat (cm/s)	0.98 (0.89, 1.10)	0.77	0.98 (0.91, 1.06)	0.62	0.97 (0.91, 1.03)	0.27
Aʹsp (cm/sec)	0.96 (0.85, 1.09)	0.55	1.03 (0.89, 1.19)	0.91	1.00 (0.89, 1.13)	1.00
Aʹlat (cm/sec)	0.93 (0.81, 1.07)	0.33	1.09 (0.99, 1.24)	0.09	0.97 (0.85, 1.10)	0.60
E/eʹsp	1.38 (1.19, 1.61)	< 0.001	1.01 (0.91, 1.12)	0.87	1.15 (1.03, 1.30)	0.02
E/eʹlat	1.27 (1.10, 1.47)	< 0.001	1.02 (0.92, 1.13)	0.74	1.11 (0.99, 1.26)	0.07
E/eʹ average	1.37 (1.17, 1.60)	< 0.001	1.01 (0.91, 1.14)	0.81	1.15 (1.01, 1.31)	0.03
LA_PLAX (mm)	0.99 (0.95, 1.03)	0.69	1.00 (0.96, 1.03)	0.84	0.99 (0.95, 1.02)	0.51
LAVI (cm^3^/m^2^)	0.99 (0.96, 1.01)	0.22	1.00 (0.99, 1.24)	0.97	1.03 (0.98, 1.08)	0.28
RVEDD (mm)	0.97 (0.91, 1.03)	0.29	1.01 (0.92, 1.03)	0.37	0.98 (0.92, 1.03)	0.39
TAPSE (mm)	0.95 (0.90, 1.00)	0.05	1.01 (0.95, 1.07)	0.85	0.97 (0.92, 1.02)	0.22
RA_4CH (cm^2^)	0.98 (0.92, 1.05)	0.60	0.96 (0.89, 1.03)	0.23	0.97 (0.91, 1.03)	0.39
TR_Vmax (m/s)	4.71 (1.24, 18.11)	0.02	2.28 (0.86, 6.46)	0.10	2.66 (0.91, 8.36)	0.08
Multivariable (forward, binary logistic regression)						
Model 1	Chi^2^ 9.18; *p* = 0.002 E/e’ average: 1.45 (1.12, 1.87), *p* = 0.005		Chi^2^ 14.78; *p* < 0.001 LVEDd: 0.91 (0.86, 0.96), *p* < 0.001		Chi^2^ 17.17; *p* < 0.001 LVEDd: 0.91 (0.86, 0.96), *p* < 0.001	
Model 2	/		Chi^2^ 4.17; *p* = 0.041 LVEDd: 0.91 (0.86, 0.96), *p* < 0.001 NTproBNP: 1.01 (1.00, 1.01), *p* = 0.047		Chi^2^ 6.99; p-0.008 LVEDd: 0.91 (0.86, 0.96), *p* < 0.001 Dec T (ms)0.99 (0.98, 0.996), *p* = 0.002 LA_PLAX (mm) 1.08 (1.02, 1.15), *p* = 0.01	

Adjustments for multiple testing were made using FDR.

Significant values are given in bold.

*OR* odds ratio, *CI* confidence intervals.

## Discussion

**Fig. 3:**
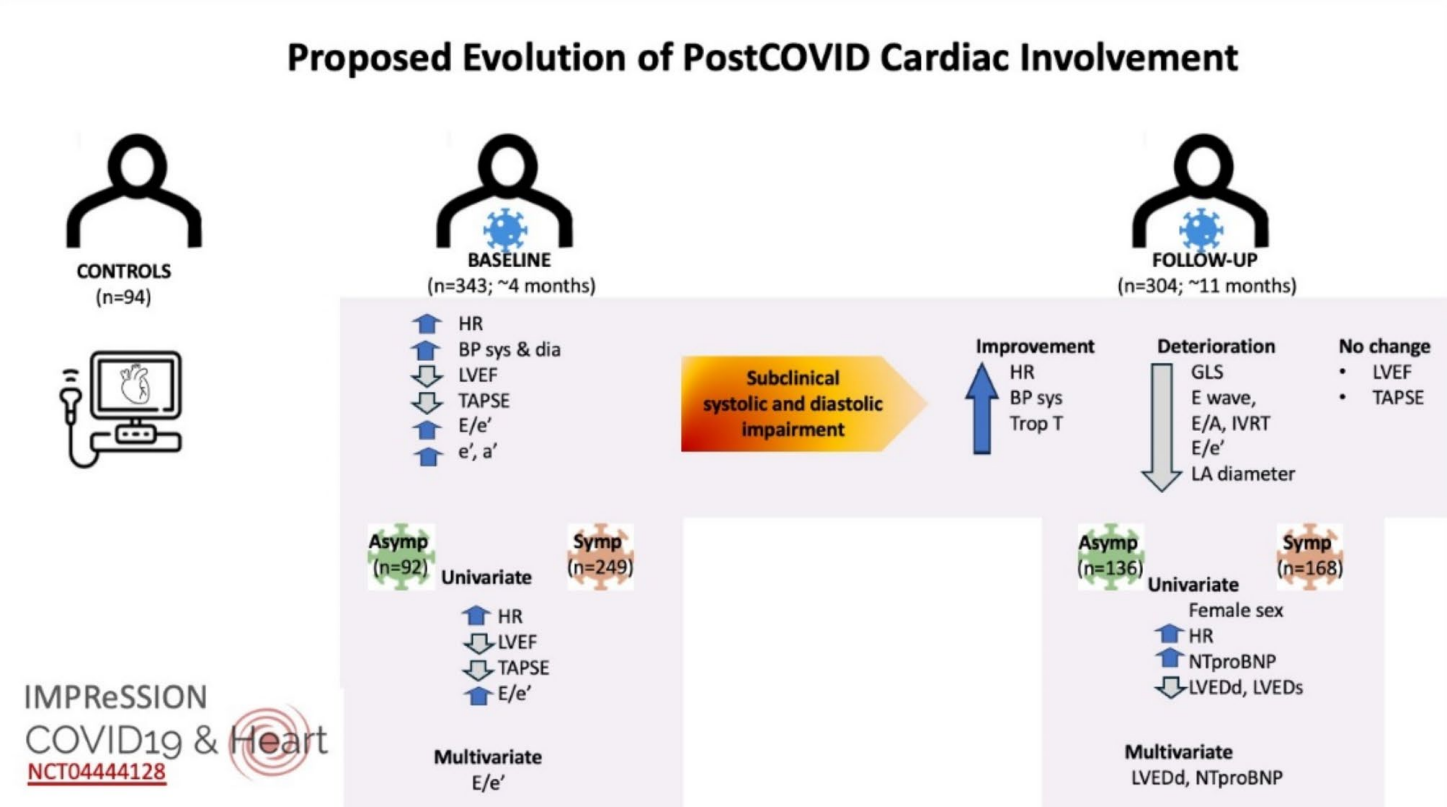
Summary of the main differences between COVID-19-infected participants and controls who underwent serial transthoracic echocardiography (TTE) and blood sampling on average at 4 and 11 months after COVID-19 infection.

In the present cohort of COVID-19-infected individuals with mild initial illness and no significant prior comorbidities, serial TTE assessments were performed to elucidate the patterns and evolution of cardiac abnormalities after COVID-19 infection. We demonstrated the presence of mild, subclinical yet significant differences in systolic and diastolic function between infected individuals and controls. Compared with those at baseline, there was a reduced systolic GLS, worsening diastolic function, and no change in LVEF or TAPSE at follow-up. Symptomatic individuals had higher heart rates at baseline and follow-up. At baseline, symptomatic individuals had a lower LVEF and more pronounced diastolic changes than did those without cardiac symptoms. At follow-up, symptomatic individuals had higher NTproBNP and lower LVEDd than did those without symptoms (Fig. 3). All the measured differences were small in magnitude and unlikely to be detectable clinically at the individual level.

This included a selected population of individuals with prior documented cases of COVID-19 who were free of preexisting cardiac conditions or significant comorbidities. Thus, none of the COVID-19-infected subjects had detectable structural heart disease, defined by increased LV wall thickness or volumetric parameters, significantly impaired LV or RV function, or significant valvular pathology, necessitating guideline-directed treatment. However, compared with controls, we detected subclinical changes in global biventricular systolic function, including LVEF and TAPSE, in COVID-19-infected individuals. The lower global biventricular function at baseline than in controls is an important finding, which is consistent with prior observations at similar time points (~ 3 months) after infection^6–10,15^, demonstrating a reduced LVEF yet with average values within the normal-preserved range^11^. Additionally, in the present study, GLS was not significantly different from that of controls at baseline; however, there was a detectable decrease in GLS at follow-up compared with the baseline value. This observation is consistent with the observations of Lassen et al., who reported that approximately 34% of previously hospitalized COVID-19 patients had impaired GLS at follow-up^10^. Furthermore, we demonstrated mild yet significant changes in TTE measures of diastolic function in infected individuals compared with controls, as indicated by higher E/eʹ and lower myocardial velocities at baseline, which is concordant with previous studies^16,17^. Although the participants in the previous cohorts differed considerably in that they mostly recovered hospitalized patients with ongoing symptoms and preexisting comorbidities, including ischaemic heart disease^14^, these observations nonetheless show that both nonhospitalized and hospitalized COVID-19-recovered individuals may share commonalities in terms of functional deterioration.

The predictive associations with cardiac symptoms suggest that the pathophysiological observations may at least in part explain the cardiac symptoms that were not present prior to the infection. The important role of diastolic function in pathophysiology is supported by the independent association of E/eʹ with symptoms at baseline, associations with biomarkers (CRP and NTproBNP), and a significant increase in E/eʹ at follow-up compared with baseline. Whereas at baseline, the drive of E/eʹ change appears to be related to reduced myocardial relaxation velocities, the increase in E/eʹ at follow-up seems influenced primarily by higher E (and lower A) mitral inflow velocities, likely reflecting higher diastolic filling pressures, which are also paralleled by an increase in LA diameter. Unlike the associations with cardiovascular symptoms at baseline, symptoms were associated with female sex, NTproBNP and smaller LV diameters at follow-up. This finding is interesting and may be related, on the one hand, to symptomatic accommodation and, on the other hand, deconditioning, which is often observed in the presence of silent cardiovascular risk factors (CVRFs), such as hypertension^18,19^ or metabolic syndrome^20^. Compared with previous work, Maestrini et al. reported no association between diastolic function at one month after hospitalization and poor outcomes at one year^21^, whereas Tudoran et al. reported impaired diastolic function in approximately 25% of patients on average at two months after index hospitalization in a relatively young cohort of patients without preexisting cardiac conditions^15^. More recently, an increased incidence of COVID-19-related hypertension driven by an increase in diastolic blood pressure was noted^22,23^, which corresponds to our own previous reports^5^ as well as the observations of the present analysis, where no improvement in diastolic pressure was noted at follow-up. An increase in aorto-ventricular stiffness was also reported in previously healthy women with mild initial COVID-19, interestingly amplified by the coexistence of metabolic syndrome^23,24^. Whereas the contribution of COVID-19 to diastolic function may be difficult to distinguish in the general population because of the overall high prevalence of CVRF, our findings in previously healthy individuals support a putative role of COVID-19 infection in accelerating diastolic impairment. TTE measures may serve as sensitive markers of early pathological findings in the aftermath of, and which are, at least in part, attributable to, COVID-19 infection.

Previous reports on the outcomes of RV function have been variable. Catena et al. reported that the TAPSE was within the normal range^25^, whereas Moody et al. reported persistently reduced RVEF in a prospective cohort study^26^. In contrast, Lassen et al. reported recovery of RV function in most patients at the 3 month follow-up^27^. We expand on these previous observations by first demonstrating that the reduction in TAPSE was more pronounced in symptomatic participants, and second, in line with a previous suggestion, there was a lack of improvement at follow-up^26^.

To our knowledge, this is the first study that looked at echocardiography findings in subgroups with cardiac symptoms versus asymptomatic participants. We previously reported that cardiac symptoms are associated with mildly reduced global systolic function and signs of mild diffuse but persistent perimyocardial inflammation detected by cardiovascular magnetic resonance^5^. We expand these previous findings by integrating a multimodality imaging approach and cardiac biomarkers. The associations of mild functional (systolic and diastolic) impairment with cardiac biomarkers at baseline are complementary and possibly instructive. It provides pathophysiological parallels with model diseases of chronic inflammation and cardiac involvement, such as systemic lupus erythematosus, cardiotoxic chemotherapy or chronic postviral syndromes^28–30^. Additionally, post-COVID-19 inflammatory involvement tends to assume a chronic indolent course, separating it from infarct-like myocarditis resulting from direct viral injury^31^.

A few limitations apply. As measures of cardiac imaging methods and biomarkers evolve in the subclinical range, the subtle findings of the current study may inform pathophysiology yet preclude their practical application in everyday clinical use. Similarly, the comparison of proportions of subjects with abnormal observations across various studies is complicated owing to the differing postprocessing methods and reporting thresholds for LVEF, GLS and E/eʹ; hence, it was omitted from the current analyses to avoid overinterpretation. We have possibly underestimated RV deterioration, as tissue tracking of the RV was not performed. The lack of echocardiographic measurements in the immediate acute phase of COVID-19 precludes the observation of the immediate effects of the infection on cardiac function. Consistent with the study hypothesis, the aim of the present study was to track the evolution of inflammatory cardiac involvement in patients who recovered after mild COVID-19.

## Data Availability

Data availability. Owing to the overall small number of patients included, there is a considerable risk of inadvertent individual identification; therefore, the dataset is not freely available. The sharing of data may be considered within the scope of ethical approval upon reasonable request to the corresponding author.

## Abbreviations

aʹ: Atrial mitral annular tissue Doppler velocity
E: Peak early diastolic transmitral flow velocity
A: Peak late diastolic transmitral flow velocity
COVID-19: Coronary virus disease 2019
E/A: Ratio of E to A
eʹ: Peak early diastolic mitral annular velocity
E/eʹ: Ratio of early diastolic filling pulsed wave Doppler to early diastolic mitral annular tissue Doppler velocity
GLS: Global longitudinal strain
HsTnT: High sensitive troponin T
IVRT: Isovolumic relaxation time
LV: Left ventricle
LVEDV: Left ventricular end-diastolic volume
LVEF: Left ventricular ejection fraction
LAVI: Left atrial volume index
MAPSE: Mitral annular plane systolic excursion
NTproBNP: N-terminal pro–B-type natriuretic peptide
RV: Right ventricle
Sa: Systolic mitral annular tissue Doppler velocity
TAPSE: Tricuspid annular plane systolic excursion
GLS: Global longitudinal strain
TTE: Transthoracic echocardiography
TR: Tricuspid regurgitation
